# Nosocomial transmission of influenza: A retrospective cross‐sectional study using next generation sequencing at a hospital in England (2012‐2014)

**DOI:** 10.1111/irv.12679

**Published:** 2019-09-19

**Authors:** Ruth M. Blackburn, Dan Frampton, Catherine M. Smith, Ellen B. Fragaszy, Simon J. Watson, R. Bridget Ferns, Špela Binter, Pietro G. Coen, Paul Grant, Laura J. Shallcross, Zisis Kozlakidis, Deenan Pillay, Paul Kellam, Stéphane Hué, Eleni Nastouli, Andrew C. Hayward, the ICONIC group, Jane Kinghorn, Fatima Wurie, Saadia Rahman, Anne Johnson, David Dunn, Andrew Leigh‐Brown, Steven Morris, William Irving, Duncan Clark, Maria Zambon

**Affiliations:** ^1^ Institute of Health Informatics UCL London UK; ^2^ Division of Infection and Immunity UCL London UK; ^3^ Department of Infectious Disease Epidemiology Faculty of Epidemiology and Population Health London School of Hygiene and Tropical Medicine London UK; ^4^ Wellcome Trust Sanger Institute Wellcome Trust Genome Campus Hinxton UK; ^5^ Clinical Microbiology and Virology University College London Hospitals NHS Foundation Trust London UK; ^6^ Infection Control Department University College London Hospitals NHS Foundation Trust London UK; ^7^ International Agency for Research on Cancer World Health Organization Lyon France; ^8^ Africa Health Research Institute Durban South Africa; ^9^ Department of Population, Policy and Practice UCL Institute of Child Health London UK; ^10^ Institute of Epidemiology and Health Care UCL London UK

**Keywords:** cross infection, disease outbreaks, influenza, human, molecular epidemiology

## Abstract

**Background:**

The extent of transmission of influenza in hospital settings is poorly understood. Next generation sequencing may improve this by providing information on the genetic relatedness of viral strains.

**Objectives:**

We aimed to apply next generation sequencing to describe transmission in hospital and compare with methods based on routinely‐collected data.

**Methods:**

All influenza samples taken through routine care from patients at University College London Hospitals NHS Foundation Trust (September 2012 to March 2014) were included. We conducted Illumina sequencing and identified genetic clusters. We compared nosocomial transmission estimates defined using classical methods (based on time from admission to sample) and genetic clustering. We identified pairs of cases with space‐time links and assessed genetic relatedness.

**Results:**

We sequenced influenza sampled from 214 patients. There were 180 unique genetic strains, 16 (8.8%) of which seeded a new transmission chain. Nosocomial transmission was indicated for 32 (15.0%) cases using the classical definition and 34 (15.8%) based on genetic clustering. Of the 50 patients in a genetic cluster, 11 (22.0%) had known space‐time links with other cases in the same cluster. Genetic distances between pairs of cases with space‐time links were lower than for pairs without spatial links (*P* < .001).

**Conclusions:**

Genetic data confirmed that nosocomial transmission contributes significantly to the hospital burden of influenza and elucidated transmission chains. Prospective next generation sequencing could support outbreak investigations and monitor the impact of infection and control measures.

## 1 BACKGROUND

Nosocomial influenza is associated with increased length of hospital stay, severe complications and death.^1^ The extent of transmission in hospital settings is poorly understood, however, because identification of transmission events is challenging. Classical methods assume cases to be “hospital‐acquired” when the time between admission and the first positive sample exceeds the incubation period of the influenza virus. This definition is not always accurate as the incubation period is variable (0.7 to 2.8 days),^2^ early symptoms may not be recorded or recognised as influenza, samples may not be taken at consistent time points within an illness, and systems often fail to capture information on hospital contact prior to admission.

Next generation sequencing methods have the potential to improve the precision of these inferences by providing information on the genetic relatedness of viral strains.^3^ Genetic approaches use assumptions about the rate at which the virus acquires mutations and the likely duration of an outbreak to assess whether direct links between patients are plausible. Availability of near real‐time sequencing data therefore raises the opportunity for improved surveillance through earlier identification of outbreaks and more effective response. Used retrospectively, information derived from next generation sequencing may also inform policy and practice for future outbreaks.

Previous applications of next generation sequencing of influenza have included elucidating zoonosis and describing transmission of seasonal and pandemic strains.^3^, ^4^, ^5^, ^6^ In the context of nosocomial transmission, several studies have used next generation sequencing to assess differences between sequences of specific influenza genome segments (HA, NA and/or PB2) or to investigate small outbreaks.^7^, ^8^, ^9^, ^10^, ^11^, ^12^, ^13^, ^14^, ^15^, ^16^, ^17^, ^18^ These results have highlighted the importance of multiple introductions of community strains. Whole genome sequencing has been used in other studies to demonstrate that isolates in pre‐defined epidemiological clusters are more likely to be related than those outside of such clusters and to differentiate outbreaks into clusters.^19^, ^20^, ^21^ However, we are unaware of studies using the greater resolution afforded by next generation sequencing of the entire genome to explore nosocomial transmission of influenza across whole seasons. Implementation of next generation sequencing has also been limited by lack of analytical capacity, absence of established quality control comparators and cost.^22^

In this study, we conducted whole genome next generation sequencing on all samples of influenza taken at a large teaching hospital in London over two winter seasons. We aimed to investigate the capability of this method to enhance identification of hospital transmission of influenza compared to methods based on routinely collected data alone and to describe transmission within the hospital setting.

## 2 METHODS

### 2.1 Study design and setting

This was a retrospective cross‐sectional study of patients at University College London Hospitals NHS Foundation Trust (UCLH). UCLH is a major teaching and research hospital in central London, which has approximately 900 beds, sees on average more than one million outpatients, has 131 000 accident and emergency attendances and admits more than 170 000 patients each year.^23^, ^24^

All laboratory‐confirmed (PCR‐positive) influenza samples taken between 13 September 2012 and 22 March 2014 were included in the study. Samples were obtained through routine care based on clinical suspicion of influenza (no formal case definitions were used to guide sampling) from inpatient, outpatient and emergency department settings. Patient demographics (age and sex), and dates of positive samples, admission, discharge and transfer between hospital wards were extracted retrospectively from electronic records. Samples from the same patient taken within a 14‐day period were assumed to be a continuation of the same illness.

### 2.2 Next generation sequencing and phylogenetic analysis

RNA was extracted from residual diagnostic specimens and sequenced using Illumina MiSeq paired‐end sequencing as previously described.^4^ Full details of phylogenetic methods are provided in the supplementary appendix. In summary, we generated consensus sequences from short reads using an in‐house de novo assembly pipeline, applying a read depth cut‐off of ≥20 reads to the final sequences. Sets of segments were compiled after categorising samples by lineage (A/(H1N1) pdm09, A/H3N2, B/Yamagata) and season (2012‐13, 2013‐14). Maximum‐likelihood phylogenetic trees were inferred for each alignment.

We defined genetic distance as the number of pairwise nucleotide differences between aligned sequences of the same subtype and within the same season. The maximum expected number of substitutions between pairs of samples was calculated using the upper bound of the 95% credibility interval of the rate of substitution and sequencing error rate for each season and lineage, assuming an upper limit of 20 days between transmission pairs and normalising for pairwise alignment length (see Table S2 for rates of substitution and sequencing error).

We defined genetic clusters as viral genomes that differed by less than the maximum expected number of nucleotide substitutions obtained from samples collected within 20 days of each other. We calculated the number of distinct genetic strains, the proportion of cases that seeded a new transmission chain (ie clusters of at least two cases) and the median number of cases per cluster.

### 2.3 Identification of nosocomial transmission

We identified potential instances of nosocomial transmission using a classical method (based on routinely collected hospital data only) and a genetic method (using results from next generation sequencing). In the “classical” method, we defined cases as hospital‐acquired if the positive sample was taken more than two days after admission and as community‐acquired if taken within 2 days. We calculated the proportion assumed to be nosocomially acquired using the formula: number of cases with the first positive sample taken more than 2 days after admission/ total number of cases.

In the “genetic” method, we considered that cases within the same genetically defined cluster were linked through transmission. We calculated the proportion assumed to be nosocomially acquired using the formula: (number of cases in genetic clusters – number of unique genetic clusters)/ number of cases. This assumes that each genetic cluster has one community‐acquired index case.

We hypothesised that cases in this hospital classified as hospital‐acquired by the genetic definition would be more likely to be hospital‐ than community‐acquired (according to the “classical” definition). We therefore calculated the proportions in each group and compared them using Fisher's exact test.

### 2.4 Identification of space‐time links

We sought to establish the extent to which pairs of cases with space‐time links based on dates and ward locations also shared genetic links. We identified space‐time links between pairs of cases with the same influenza subtype based on their assumed infectious and “acquisition” periods (Figure S1). The acquisition period was the period in which they may have been infected and was derived from the incubation period (1‐3 days) plus an interval (0‐2 days) between onset of symptoms and sample collection.^2^ Acquisition periods therefore ranged from 1 to 5 calendar days prior to the sample collection date. We considered acquisition to be possible in the hospital ward where the sample was taken and all wards where the patient was treated during the assumed acquisition period. We defined the infectious period as lasting a maximum of 14 days starting from two days before the sample date.^25^ We also conducted sensitivity analyses varying the length of the infectious period (Appendix S1).

Pairs of cases were classified as having space‐time links if they had the same influenza subtype and overlapping infectious and acquisition periods whilst in the same hospital location. We calculated the proportion of cases in genetic clusters that had space‐time links with cases in the same genetic cluster (and therefore also of the same influenza subtype). We also hypothesised that pairs of cases with space‐time links would have closer genetic links than pairs of cases that were linked temporally (ie by overlap in infectious and acquisition periods) but did not have spatial co‐occurrence. Time‐linked cases were used for this comparison to account for the accumulation of independent genetic changes over time. We investigated this by comparing the genetic distances (regardless of cluster assignment) amongst these pairs of cases with the Wilcoxon rank‐sum test.

Finally, we combined epidemiological and genetic data to visualise potential transmission links. Data were managed, analysed and visualised using Stata v14 and R v3.5.0.

### 2.5 Ethical approval

REC approval (13/LO/1303) for ICONIC was received on 20th August 2013, IRAS project ID 131373. Approval applies to all NHS sites taking part in the study and additional permissions have been obtained from the NHS/HSC R&D offices of all partner sites prior to the start of the study.

## 3 RESULTS

A total of 332 PCR‐positive influenza samples were identified during the study period. Full genome sequencing was possible for 242 (72.9%) samples, from 214 patients. It is likely that sequencing was not successful for the remaining samples due to insufficient viral load. All subsequent analyses are based on the samples for which sequencing was successful. The characteristics of the patient population are shown in Table 1.

**Table 1:** Characteristics of patients with influenza samples sequenced by full genome sequencing, University College London Hospitals NHS Foundation Trust, 2012‐2014 (n = 214)

	Number of patients	%
Total	214	–
**Gender**		
Male	120	56.1
Female	94	43.9
**Age group (y)**		
<5	38	17.8
5‐14	18	8.4
15‐64	119	55.6
65+	39	18.2
**Sample collection date**		
September 2012‐August 2013	183	85.5
September 2013‐March 2014	31	14.5
**Sample collection location**		
Inpatient ward	115	53.7
Outpatient clinic	23	10.7
Accident and Emergency	76	35.5
**Duration of hospital admission (amongst 132 admitted patients, d)**		
Median	5	–
Interquartile range	5‐12	–
**Influenza subtype**		
A H3N2	82	38.3
A H1N1	52	24.3
A	18	8.4
B	62	29.0

Phylogenetic trees for each influenza subtype (influenza A H3N2, influenza A (H1N1) pdm09 and influenza B Yamagata‐like) are shown in the appendix (Figure S2). There were 180 unique strains, of which 16 (8.9%, approximately 1 in 11) seeded a new transmission chain. The remaining 164 strains did not cluster with other cases. The 16 genetic clusters included 50 patients in total, with a median cluster size of 3 (range 2‐5). Although not a pre‐requisite for forming clusters, the majority (44/50) of clustered samples were supported by a bootstrap confidence value of greater than 75% in the phylogeny and 42/50 by a value of greater than 90%.

Using the classical definition of nosocomial transmission, 15.0% (32/214) cases were classified as hospital‐acquired (tested positive for influenza more than two days after admission). Using the genetic definition, 15.8% (34/214) cases were classified as due to nosocomial transmission (50 cases in genetic 16 clusters, 16 index cases assumed to be introduced from community and 34 due to onward transmission). The concordance between these methods is shown in Table 2: The hospital‐acquired cases (according to the classical definition) were more likely to be classified as hospital‐acquired by the genetic method (19/32, 59.4%) than community‐acquired cases (31/182, 17.0%, *P* < .001).

**Table 2:** Evidence for nosocomial transmission of influenza using classical and genetic methods, University College London Hospitals NHS Foundation Trust, 2012‐2014 (n = 214)

Classical definition^a^	Genetic definition^b^			
	Hospital‐acquired (n = 34)		Community‐acquired (n = 180)	
	n	%^c^	n	%
Hospital‐acquired (n = 32)	14	43.8	18	56.3
Community‐acquired (n = 182)	20	11.0	162	89.0

a Cases defined as hospital‐acquired if PCR‐positive influenza sample was taken more than two days after admission; and as community‐acquired if taken within two days.

b Cases defined as hospital‐acquired if they were part of a genetically defined cluster (except the first case to be identified in the cluster, classified as the “index” case); and as community‐acquired if they were index cases or had unique genetic strains.

c Row percentages

**Figure 1:**
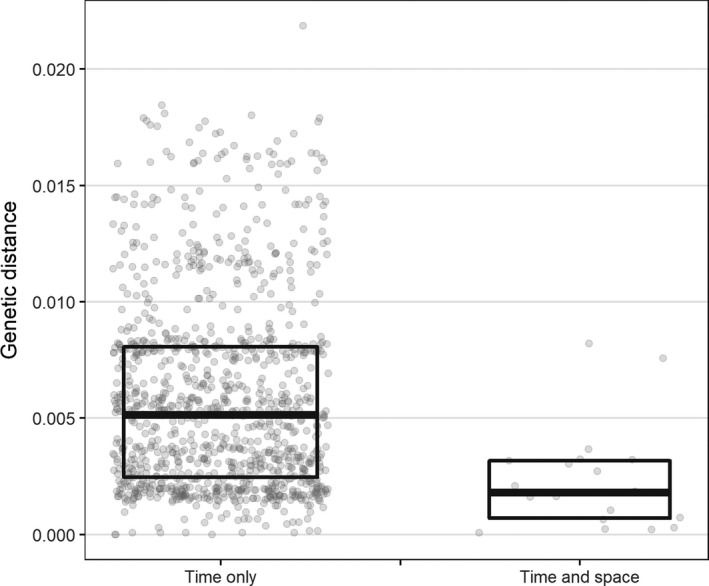
Normalised genetic distances between pairs of influenza H1N1 and H3N2 cases that were linked in time and space, University College London Hospitals NHS Foundation Trust, 2012‐2014 (n = 134). Time only: Links between cases based on overlapping assumed infectious and acquisition periods only. Space‐time: Links between cases based on overlapping assumed infectious and acquisition periods whilst in the same hospital location

Of the 50 cases in genetic clusters, 11 (22.0%) had space‐time links (based on routinely collected data) with other cases in the same genetic cluster. Genetic distances between pairs of cases that had space‐time links were smaller (median 1.8 × 10^−3^ substitutions/site, interquartile range 0.7‐3.1) than between pairs of cases that were linked in time only (5.1 × 10^−3^ substitutions/site, interquartile range 2.5‐8.1, *P* < .001; Figure 1).

**Figure 2:**
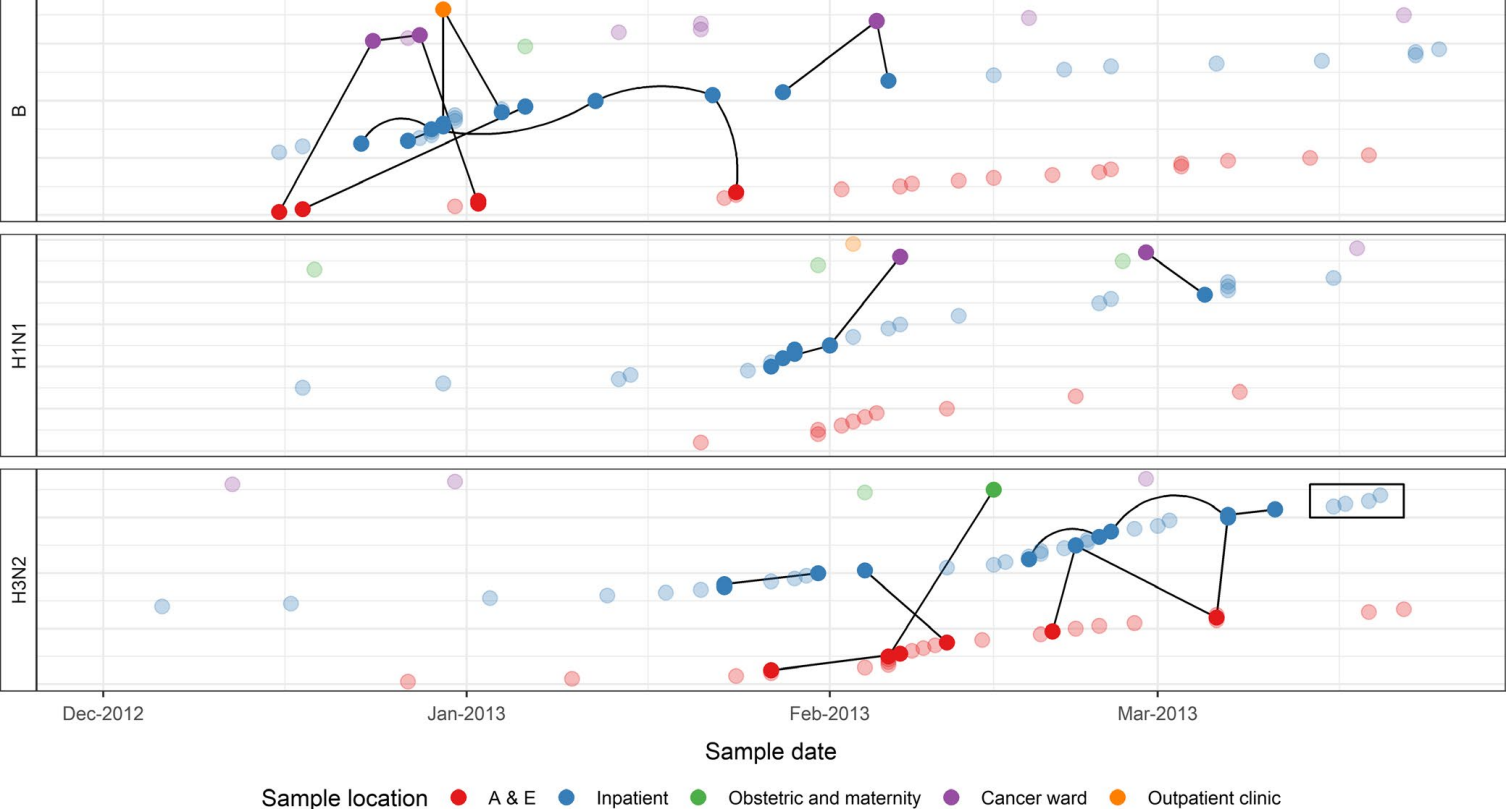
Space‐time links between cases of influenza, University College London Hospitals NHS Foundation Trust, 2012‐2013 (n = 183). Dots show cases on the date of their first positive influenza specimen. Solid dots indicate cases that were part of genetic clusters, and transparent dots were not clustered. Black lines indicate genetic links between cases. The colour of the marker indicates the hospital area where the sample was taken. The rectangle highlights a group of cases for which transmission may have been suspected based on time and place of sampling, but is not indicated by genetic data. In this figure, only the cases H1N1 cases in the Inpatient ward were sufficiently close in time to be classified as having space‐time links

Space‐time and genetic links between cases (first sample per person only) are displayed visually for the 2012‐2013 influenza season in Figure 2. This figure highlights that cases (dots) with genetic links (black lines) are frequently sampled on the same ward (colour of dot) at around the same time. It also shows instances where transmission links may have been presumed (patients on the same ward at around the same time, eg cases in rectangle), but genetic data show the cases are not part of the same transmission chain.

## 4 DISCUSSION

We have used whole genome sequencing, on an established next generation sequencing platform, to investigate nosocomial spread of influenza across two winter seasons. Based on genetic data, we found that one in eleven cases of influenza introduced to a hospital seeded a new transmission chain. This resulted in an average of three instances of presumed onward transmission, with at least 16% of the total cases of influenza in the hospital due to nosocomial transmission. Cases resulting from these presumed transmission events were more likely to meet the classical definition of nosocomial infection (occurring two or more days after admission to the hospital than within two days), (*P* < .001). This supports the capability of virus genetics to be used to identify nosocomial transmission.

Our estimate of the extent of nosocomial transmission based on the classical method (15%) was similar to previous estimates in UK settings, which defined nosocomial infection based on presentation of symptoms 3‐4 days after hospital admission and gave estimates of nosocomial infection of 2%‐12%.^1^, ^26^ Although our estimate derived using genetic clustering produced a similar value (16%), there was a lack of concordance between the two methods. Only 14/32 (44%) cases classified as hospital‐acquired by the classical method were classified as hospital‐acquired using the genetic method, and 14/34 (41%) cases classified as hospital‐acquired by the genetic method were hospital‐acquired according to the classical method. The accuracy of the classical method is limited by lack of symptom onset information and variation in sampling practice. For example, if a patient has symptoms of influenza when they are admitted to hospital, but are not sampled within two days, they would be incorrectly classified as having hospital‐acquired infection, but may be shown not to be in a genetic cluster leading to discordant results. The genetic method does not rely on these assumptions, and can establish direct transmission links between cases, and is therefore likely to be more reliable. It is also possible that cases classified as hospital‐acquired by the genetic method within two days of admission were part of community clusters, but this is unlikely given the diversity of community strains.

Pairs of cases that had space‐time links (derived from dates and ward locations recorded in routine hospital data) had smaller genetic distances than those without space‐time links (*P* < .001). This indicates closer genetic relatedness and is consistent with studies in household, hospital and long‐term care facility settings.^19^, ^20^, ^27^, ^28^, ^29^ However, only 22% of cases in genetic clusters had space‐time links with other cases in the same genetic cluster. This implies that most transmission is not through obvious ward‐based contact. Genetic clustering analysis could therefore be useful to distinguish genuine outbreaks from coincidental pairs of cases on wards and to direct control efforts accordingly.

This study aimed to describe how virus genomics could improve understanding of nosocomial transmission gleaned from routine hospital data and clinical practice. As such, there was no enhanced sampling or epidemiological investigation to identify potential interactions between patients outside ward settings. Results will therefore have been based on incomplete case ascertainment, and transmission occurring on non‐ward settings, from sub‐clinically infected patients, staff members or visitors could not be detected. This demonstrates an advantage of using sequencing data, which can group cases into genetic clusters even if some of the links in the transmission chain are missing. Enhanced sampling of patients, staff and visitors to identify all cases and prospective collection of contact data would likely be needed to establish evidence of contact between a greater proportion of genetically clustered cases than was possible using retrospective patient ward movement data.

A limitation of our analysis is that we did not have information on symptoms, co‐morbidities or clinical outcomes such as length of stay. We therefore could not estimate which of these factors may have influenced transmission or severity of illness. We also did not have data on negative tests for influenza and were therefore unable to ascertain if individuals were tested before their positive sample was taken. However, the infection control policy in this hospital is to isolate all patients presenting with influenza‐like illness on admission until results of PCR testing are known. Another limitation was the definition of genetic clusters, which was based on small differences between genomes and would therefore be sensitive to small variations in sequences and sequencing errors. However, the analysis based on genetic distance measures did not involve grouping isolates into clusters. This analysis showed that pairs of cases had closer genetic relatedness when they had space‐time links and supports the findings from cluster‐based analyses. A previous simulation study has shown that the expected number of changes between two influenza genomes that come from a direct transmission event is likely to be 0 or 1, in line with our results.^30^

The sequencing, gene assembly, phylogenetic and epidemiological analyses presented here have the potential to be automated to provide near real‐time (within 24‐48 hours) pictures of transmission within hospitals. If implemented in multiple hospitals, both locally and internationally, the estimates could be used to inform surveillance for comparison of influenza strains in circulation and their transmission potential. It could also be used for earlier identification of outbreaks, enabling introduction of more intensive control efforts. This may include increased testing to identify, isolate and treat cases earlier, cohorting, enhanced hand hygiene, engineering approaches to increase ventilation, use of respiratory protection and vaccination of staff or vulnerable patient groups. Analyses such as we present here can also provide accurate numbers to measure changes in policy or prevention and for ascertaining best practice in different health care settings. For example, viral genetic sequencing could be used to assess the impact of healthcare worker vaccination and visitor infection control practices on the extent of transmission.

In conclusion, our results demonstrate the value of routine whole genome sequencing to inform influenza surveillance and infection control interventions in hospitals. Genetic data confirmed nosocomial transmission for approximately 16% of cases, with short chains of transmission. These results suggest that integrating next generation sequencing to real‐time investigations of influenza in hospital could inform strengthened infection control measures to minimise the burden of nosocomially acquired infection.
