# Supplementary material for: Nosocomial transmission of influenza: A retrospective cross‐sectional study using next generation sequencing at a hospital in England (2012‐2014)
Source: Influenza Other Respir Viruses. 2019 Sep 19;13(6):556–63. doi: 10.1111/irv.12679 (PMC6800305; doi:10.1111/irv.12679)
Supplement: Supplementary file 1 [file IRV-13-556-s001.docx]

**Nosocomial transmission of influenza: a retrospective cross-sectional study using next generation sequencing at a hospital in England (2012-2014)**

**SUPPLEMENTARY APPENDIX**

**ADDITIONAL METHODS**

**Genome assembly, alignments and phylogenetic analysis**

Consensus sequences were generated from short reads using an in-house de novo assembly pipeline developed by the ICONIC project, applying a read depth cut-off of ≥20 reads to the final sequences. Briefly, for each sample, reads were trimmed to remove adapter sequences and until their median Phred score was >=30 using Trimmomatic [1]. These were assembled into contigs using IVA (Iterative Virus Assembler) v1·0·0, [2] with custom scripts used to concatenate, extend and resolve overlapping sequences for each segment by iterative read mapping, correcting majority base errors after each round of mapping. Sets of segments were compiled after categorising samples by lineage (A/(H1N1) pdm09, A/H3N2, B/Yamagata) and season (2012-13, 2013-14) (Table S1). Phylogenetic analysis was undertaken firstly by aligning each set of segments using MAFFT v7·3·05 [3] and concatenating the coding regions within Aliview [4]. Maximum-likelihood phylogenetic trees were inferred for each alignment under a general-time reversible substitution model with rate heterogeneity among sites modelled under a 4-category discrete approximation of a gamma distribution using RAxML v8·2·10 [5]. Branch support was assessed through non-parametric bootstrapping of 1000 pseudoreplicates.

**Calculation of the expected number of nucleotide substitutions between pairs of linked samples**

For each influenza A lineage and season we used BEAST c1·8·4 [6] to estimate the underlying rate of nucleotide substitutions per site per year for each segment and its associated variance. For all four analyses, two MCMC chains were run for 250 million generations, logging every 10,000 states. A burn-in of 10% was used and convergence of the chains was assessed using Tracer v 1.6. [7] For all runs, the SDR06 nucleotide substitution model was used, along with a relaxed molecular clock and a Bayesian Skyline coalescent prior. We partitioned the genomic alignments by segment and thus were able to estimate rates of substitution for each segment (and associated variances). We did not use this approach for influenza B due to poor sequencing generating only partial genomes which would negatively impact tree reconstruction. In addition, the segments that were successfully sequenced are known to have higher rates of substitution than the genome as a whole and thus would over-estimate the expected number of substitutions.

We also considered the number of differences we would expect to see due to sequencing error. The rate of sequencing error per nucleotide was estimated by measuring the number of nucleotide differences between segments assembled from repeated runs of the same influenza samples from a separate project (61 samples, 142 lanes in total; some samples were sequenced more than twice; data not shown). The observed error rate was 1·14 nucleotides per genome (standard deviation 2.38nt).

We defined genetic distance as the number of pairwise nucleotide differences between aligned sequences of the same strain and within the same season. The maximum expected number of substitutions between pairs of samples was then calculated using the upper bound of the 95% credibility interval of the rate of substitution and sequencing error rate for each season and lineage, assuming an upper limit of 20 days between transmission pairs and normalising for pairwise alignment length.

**Definition of genetic clusters**

We defined genetic clusters as viral genomes that differed by less than the maximum expected number of nucleotide substitutions, and from a monophyletic cluster supported by a bootstrap score ≥95% in the phylogeny. Virus isolates also had to be collected within 20 days of each other (which extends across the incubation and infectious periods outlined in the previous section) to be considered as directly linked, i.e. representative of a direct transmission event.

We identified the number of distinct genetic clusters and calculated the median number of cases per cluster. We assumed that genetically-defined clusters consisted of an index case who introduced the infection from the community and subsequent cases who were a result of transmission in the hospital. Based on this classification, we calculated the number of cases that seeded a new transmission chain (i.e. the number of distinct clusters), and the proportion of cases that were due to subsequent nosocomial transmission.

We also explored defining clusters based on examining for a bi-modal distribution of genetic distances between pairs of samples within the same season and strain (which we had previously observed from analysis of genomic data within small hospital outbreaks). However, in this instance the distributions were not clearly bi-modal and thus we used the above method to define genetic clusters.

**Identification of space-time links: sensitivity analysis**

We conducted sensitivity analyses to ascertain the significance of varying the length of the infectious period on the identification of space-time links. In these analyses, we used maximum infectious period durations of 5, 9 and 30 days, reflecting uncertainty around the number of days of viral shedding in patients with differing levels of comorbidity and immune function [8–14].

**ADDITIONAL RESULTS**

**Phylogenetic analysis**

Sequencing and subsequent genome assembly was particularly successful for influenza A ((H1N1) pdm09, H3N2) samples generating whole genomes for the vast majority of samples across all segments (Table S1, Figures S3, S4A-B). This was not the case for influenza B samples, with the majority only successfully generating sequence for 3 segments (PB2, PB1 and PA) (Figures S3, S4C). However, read depths of coverage were good for each lineage, with median depths of coverage of 2814 and 2000 for influenza A and B respectively (Figure S5).

The estimated expected number of substitutions was small, corresponding to a mean number of differences of 1·0-1·2 nucleotides across a whole genome (see Table S2 for more details). For each lineage and season this was equivalent to ~7 nucleotide substitutions, or 5·1-5·2x10^-3^ substitutions per site per year.

**Identification of space-time links: sensitivity analysis**

In sensitivity analysis, increasing the infectious period from 14 to 30 days made no difference to the number of cases with space-time links; reducing the infectious period to 5 or 9 days resulted in one fewer case defined as having these links.

**TABLE S1**: **Assembled genome lengths across each lineage**

| **Genome lengths** | **Min.** | **Q1** | **Median** | **Q3** | **Max.** |
| --- | --- | --- | --- | --- | --- |
| H1N1 | 4928 | 13389.3 | 13540.5 | 13584 | 13610 |
| H3N2 | 1463 | 13486 | 13564 | 13609.3 | 13627 |
| B | 2275 | 6924 | 6928 | 8796 | 14397 |

**TABLE S2**: **Summary of estimated rates of substitutions per site derived from BEAST, estimated error rates of sequencing and the final cut-off used for each lineage and season**

|  |  | **Strain and season** | | | |
| --- | --- | --- | --- | --- | --- |
|  | **Measure** | **H1N1 2012-13** | **H1N1 2013-14** | **H3N2 2012-13** | **H3N2 2013-14** |
| Rate of substitutions per site per year | Mean  Standard deviation | 1.4e-03  5.7e-04 | 1.53e-03  6.42e-04 | 1.57e-03  5.04e-04 | 1.35e-03  5.34e-04 |
| Expected number of substitutions per genome in 20 days | Number of substitutions  Standard deviation | 1.05  0.42 | 1.1  0.37 | 1.15  0.46 | 0.97  0.38 |
| Expected number of sequencing errors per genome | Number of nucleotides  Standard deviation | 1.14  2.38 | 1.14  2.38 | 1.14  2.38 | 1.14  2.38 |
| Total expected number of substitutions | Number of substitutions  Standard deviation | 2.19  2.42 | 2.24  2.41 | 2.29  2.42 | 2.11  2.41 |
| Cut-off (maximum number of expected substitutions) | Upper limit of 95% confidence interval | 6.93 | 6.96 | 7.04 | 6.83 |

**FIGURE S1: Schematic depicting an influenza transmission chain of three cases where hypothetical acquisition and infectious periods for a case are determined relative to its sample date**

The overlap between the acquisition and infectious periods reflects uncertainty around the duration of the incubation period and the time of sampling relative to onset of symptoms. The index case (Case A) within the transmission chain is a potential “donor” for transmission to both “recipient” Cases B and C (provided that the patients were located on the same ward during this timeframe), which is denoted using dashed lines. Transmission from Case B to Case C is also plausible (indicated using blue arrows) given the overlap in the likely infectious period for Case B and likely acquisition period for Case C.

**FIGURE S2: Maximum-likelihood phylogenetic trees inferred from whole genome alignments for A) influenza A (H1N1) pdm09 , B) influenza A H3N2 , C) influenza B Yamagata-like lineages.**

Isolates collected in this study are shown as circles, coloured by season (red: 2012-2013; blue 2013-2014). Reference viruses for designated clades are shown in black, with the relevant clade numbering given to the right. Trees are rooted on the oldest reference isolate. Nodes with more than 70% bootstrap support are highlighted with purple circles. The scale bar is given in units of substitutions per site. Samples determined as linked by genetic distance and date are denoted with asterisks (*). Samples with identical sequences (and thus excluded from the tree) are denoted by double asterisks (**).

A)


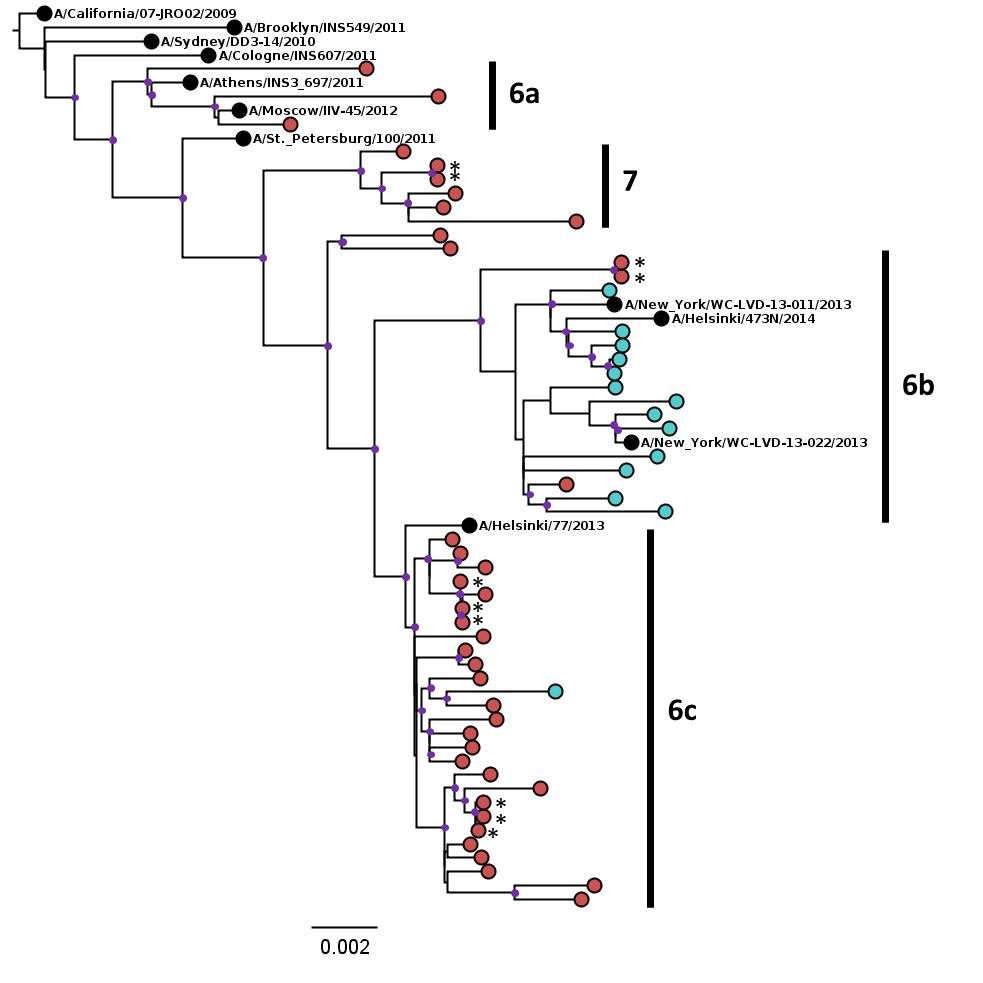


B)


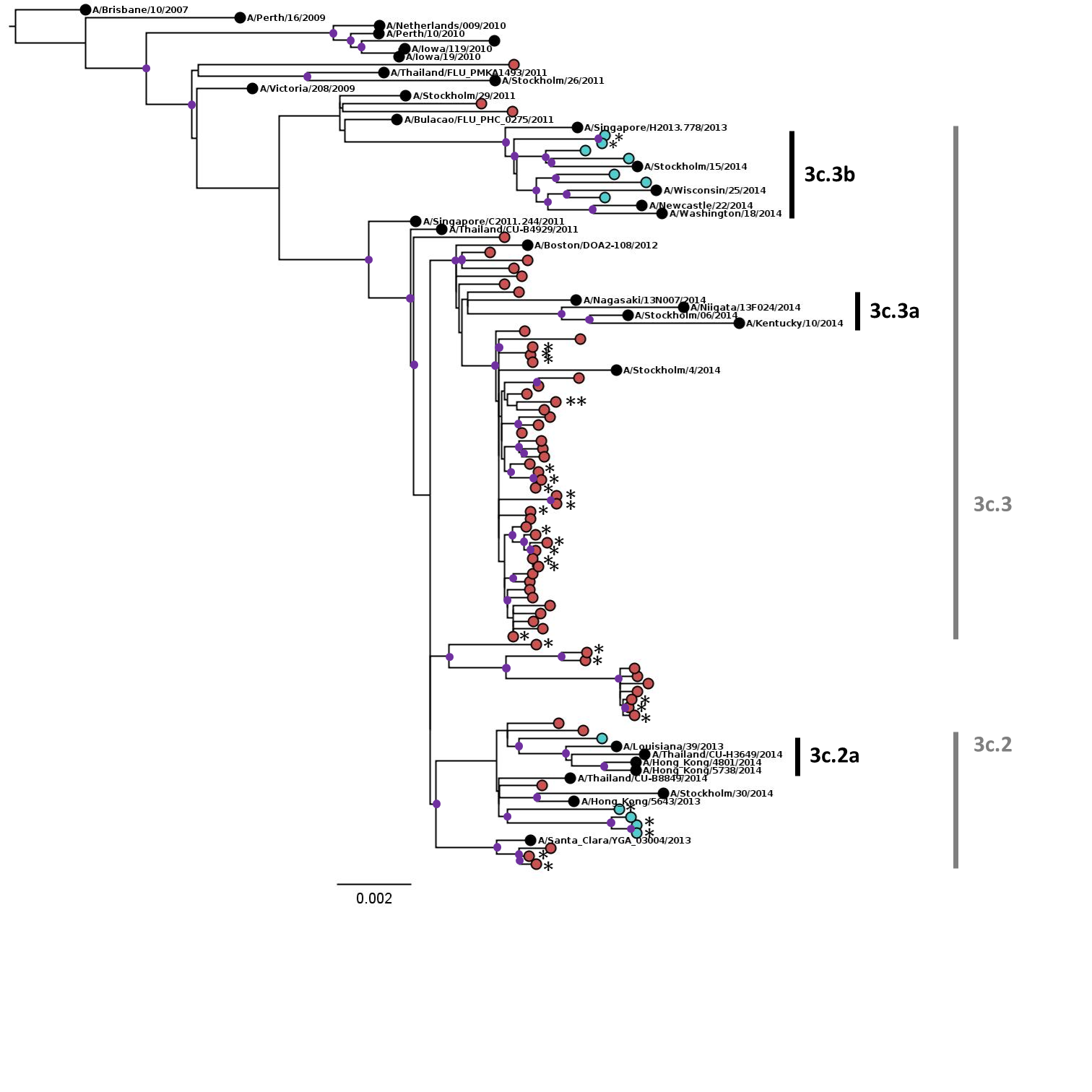


C)


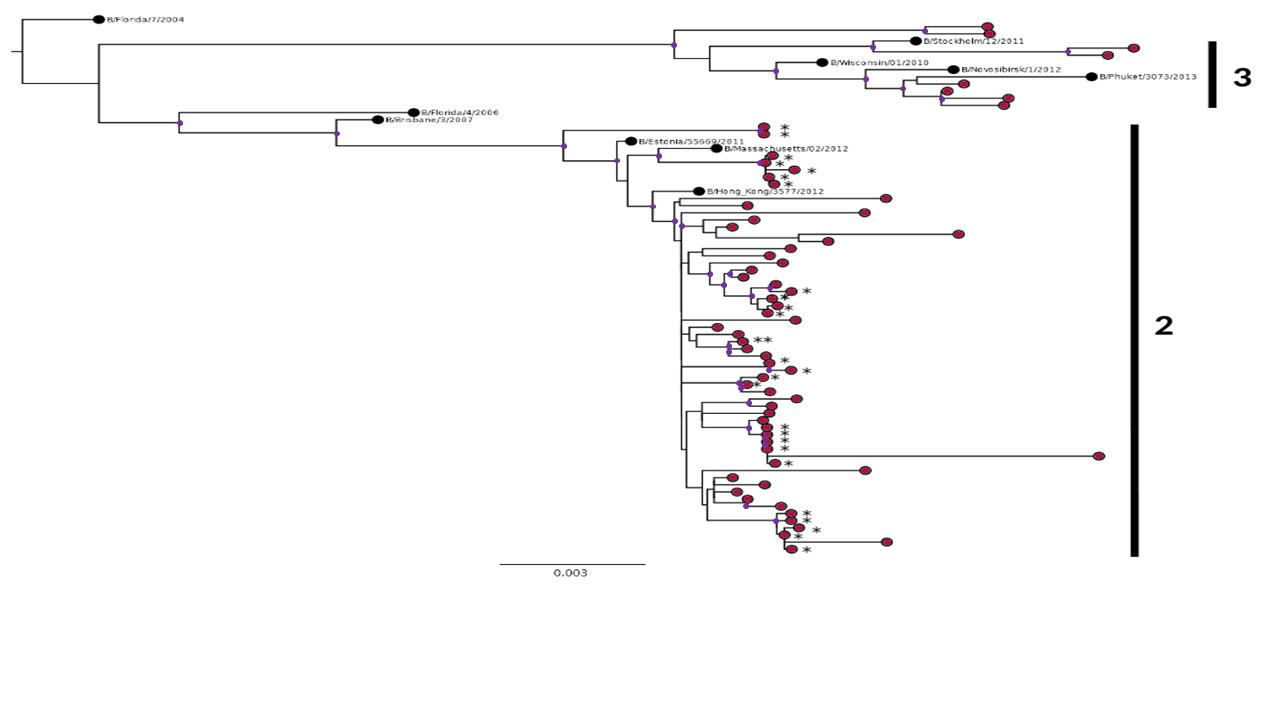


**FIGURE S3:** Boxplots illustrating assembled genome lengths for each lineage. Boxes extend to the 1^st^ and 3^rd^ quartiles, whiskers extend to 1.5 x inter-quartile range.


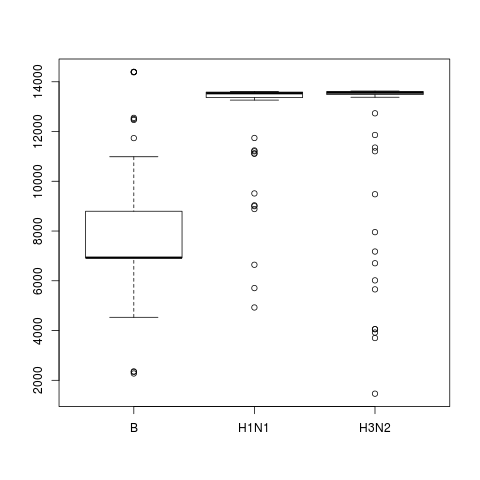


**FIGURE S4**: **Boxplots illustrating alignment coverage to representative reference sequences by segment for a) A/H1N1, b) A/H3N2 and c) B/Yamagata lineages.**

Boxes extend to the 1^st^ and 3^rd^ quartiles, whiskers extend to 1.5 x inter-quartile ranges.

A)


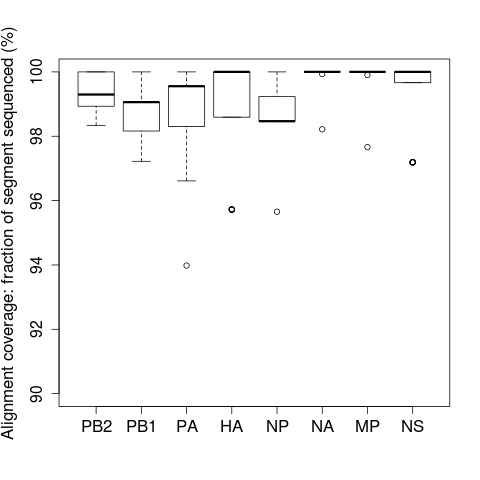


B)


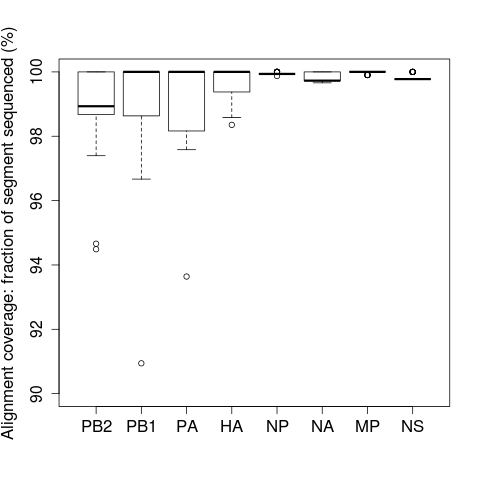


C)


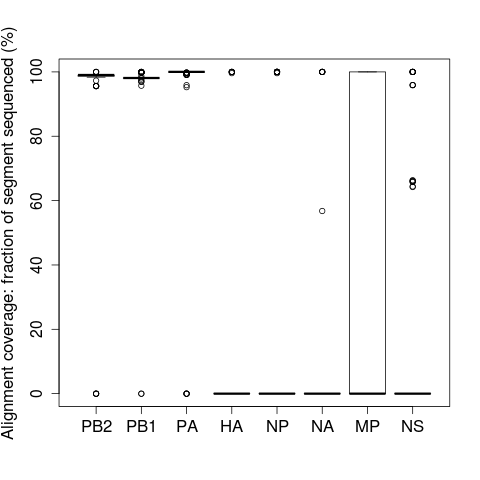


**FIGURE S5: Boxplots illustrating median read depth across each segment for a) A/H1N1, b) A/H3N2 and c) B/Yamagata lineages**

Boxes extend to the 1^st^ and 3^rd^ quartiles, whiskers extend to 1.5 x inter-quartile range.

A)

**
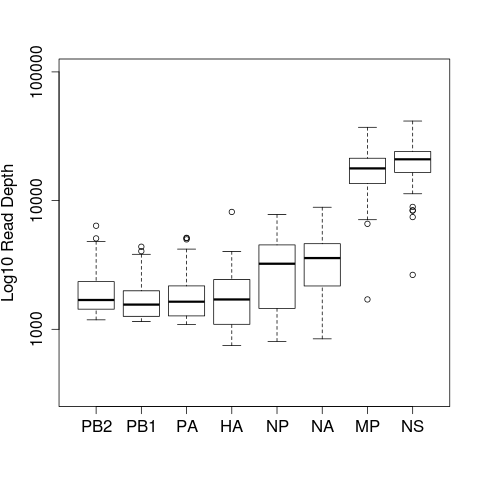
**

B)


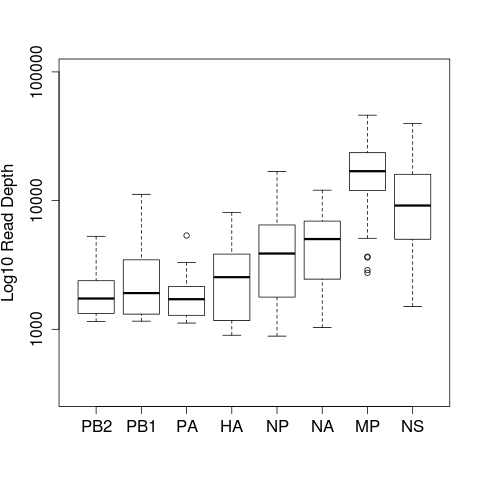


C)


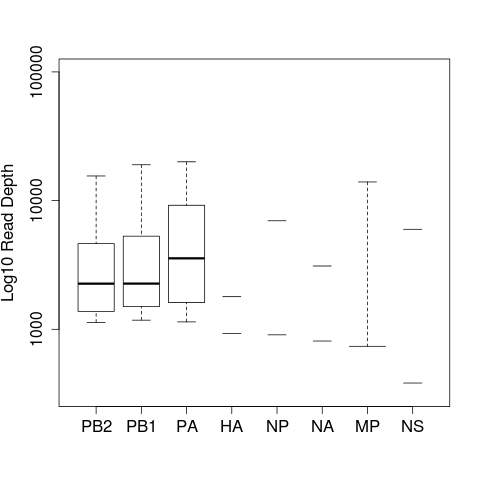


**REFERENCES**

1 Bolger AM, Lohse M, Usadel B. Trimmomatic: a flexible trimmer for Illumina sequence data. *Bioinformatics* 2014;**30**:2114–20. doi:10.1093/bioinformatics/btu170

2 Hunt M, Gall A, Ong SH, *et al.* IVA: accurate de novo assembly of RNA virus genomes. *Bioinformatics* 2015;**31**:2374–6. doi:10.1093/bioinformatics/btv120

3 Katoh K, Standley DM. MAFFT multiple sequence alignment software version 7: improvements in performance and usability. *Mol Biol Evol* 2013;**30**:772–80. doi:10.1093/molbev/mst010

4 Larsson A. AliView: a fast and lightweight alignment viewer and editor for large datasets. *Bioinformatics* 2014;**30**:3276–8. doi:10.1093/bioinformatics/btu531

5 Stamatakis A. RAxML version 8: a tool for phylogenetic analysis and post-analysis of large phylogenies. *Bioinformatics* 2014;**30**:1312–3. doi:10.1093/bioinformatics/btu033

6 Drummond AJ, Suchard MA, Xie D, *et al.* Bayesian phylogenetics with BEAUti and the BEAST 1.7. *Mol Biol Evol* 2012;**29**:1969–73. doi:10.1093/molbev/mss075

7 Rambaut A, Drummond AJ, Xie D, *et al.* Posterior summarisation in Bayesian phylogenetics using Tracer 1.7. *Syst Biol* 2018;:syy032-syy032. doi:10.1093/sysbio/syy032

8 Fielding JE, Kelly HA, Mercer GN, *et al.* Systematic review of influenza A(H1N1)pdm09 virus shedding: duration is affected by severity, but not age. *Influ Other Respir Viruses* 2014;**8**:142–50. doi:10.1111/irv.12216 [doi]

9 Carrat F, Vergu E, Ferguson NM, *et al.* Time lines of infection and disease in human influenza: a review of volunteer challenge studies. *Am J Epidemiol* 2008;**167**:775–85. doi:10.1093/aje/kwm375

10 Hayden FG, Jennings L, Robson R, *et al.* Oral oseltamivir in human experimental influenza B infection. *Antivir Ther* 2000;**5**:205–13.

11 Jao RL, Wheelock EF, Jackson GG. Production of interferon in volunteers infected with Asian influenza. *J Infect Dis* 1970;**121**:419–26.https://watermark.silverchair.com/121-4-419.pdf?token=AQECAHi208BE49Ooan9kkhW_Ercy7Dm3ZL_9Cf3qfKAc485ysgAAAcgwggHEBgkqhkiG9w0BBwagggG1MIIBsQIBADCCAaoGCSqGSIb3DQEHATAeBglghkgBZQMEAS4wEQQMuHY_d-VvZC9ObPO_AgEQgIIBe_Lk4_0l_f0Fq_GXHdTP8N4YaZnZraqacLLVibLDeChaP

12 Reuman PD, Bernstein DI, Keefer MC, *et al.* Efficacy and safety of low dosage amantadine hydrochloride as prophylaxis for influenza A. *Antivir Res* 1989;**11**:27–40.https://ac.els-cdn.com/0166354289900181/1-s2.0-0166354289900181-main.pdf?_tid=17eaf903-53c7-4cf8-a6a1-1ac6dbdf97e7&acdnat=1530547875_f9df63e2cd3b30a119c11ae7acc68a34

13 Memoli MJ, Athota R, Reed S, *et al.* The natural history of influenza infection in the severely immunocompromised vs nonimmunocompromised hosts. *Clin Infect Dis* 2014;**58**:214–24. doi:10.1093/cid/cit725

14 Weinstock DM, Gubareva L V, Zuccotti G. Prolonged shedding of multidrug-resistant influenza A virus in an immunocompromised patient. *N Engl J Med* 2003;**348**:867–8. doi:10.1056/nejm200302273480923
